# Spread of Antifungal-Resistant *Trichophyton indotineae*, United Kingdom, 2017–2024

**DOI:** 10.3201/eid3101.240923

**Published:** 2025-01

**Authors:** Alireza Abdolrasouli, Richard C. Barton, Andrew M. Borman

**Affiliations:** King's College Hospital, London, UK (A. Abdolrasouli); Leeds Teaching Hospitals National Health Service Trust, Leeds, UK (R.C. Barton); Southmead Hospital, Bristol, UK (A.M. Borman); University of Exeter, Exeter, UK (A.M. Borman)

**Keywords:** Trichophyton indotineae, dermatophytosis, tinea cruris, antifungal resistance, antimicrobial resistance, fungi, terbinafine, treatment failure, United Kingdom

## Abstract

We describe 157 cases of *Trichophyton indotineae* infection in the United Kingdom, mostly in patients linked to southern Asia. *T. indotineae* is spreading in the United Kingdom and accounts for 38% of dermatophyte isolates referred to the UK National Mycology Reference Laboratory. Clinicians should suspect *T. indotineae* in tinea corporis cases.

Outbreaks of superficial skin infections caused by the emergent dermatophyte *Trichophyton indotineae* (*Trichophyton mentagrophytes* genotype VIII) were reported in southern Asia starting in 2014 (*1*–*4*). Typically, *T. indotineae* infections initially involve the groin (tinea cruris) and respond poorly to treatment, resulting in widespread lesions affecting multiple body sites. Many isolates exhibit in vitro resistance to terbinafine, and most infections are clinically resistant to that drug (*1*–*5*). Infections spread easily from person to person (*1*–*8*), and some reports suggest sexual transmission (*9*).

*T. indotineae* is endemic across Asia, but cases have been reported worldwide (*4*), including in Europe (*5*–*7*), Canada (*8*), and the United States (*9*). Mounting evidence suggests infection acquisition and transmission outside original areas of endemicity (*5*,*7*,*9*,*10*). Occasional cases of *T. indotineae* infection have been reported from the United Kingdom (*10*). We describe all cases of *T. indotineae* identified at the UK National Mycology Reference Laboratory (MRL) during a 7-year period.

We reviewed laboratory records from August 2017–July 2024 for dermatophytes identified as *T. indotineae*. When available, we extracted clinical and epidemiologic data from requisition forms. Dermatophyte identification was determined by whole-genome sequencing (WGS) or internal transcribed spacer sequencing, combined with phenotypic identification (Appendix Table). Isolates received after 2021 were identified using phenotypic features alone. A key defining microscopic feature was abundant fusiform to clavate, thin smooth-walled macroconidia with an acute apical tip, as well as other macroscopic and microscopic characteristics (Appendix Figure 1). We performed susceptibility testing by broth microdilution according to Clinical and Laboratory Standards Institute standards (Appendix). In the absence of an established clinical breakpoint for terbinafine, we used an MIC of >0.5 mg/L to identify non–wild-type isolates.

The first WGS-confirmed case we noted was from October 2018. In nearly half (42.7%, 67/157) of identified cases, the groin, buttocks, and thighs were directly involved, and neighboring body sites (abdomen and back) were implicated in another 18 cases (Table). Most (84.7%) patients had links to endemic areas, including South Asian ethnic background (n = 97), recent travel to the Indian subcontinent or Middle East (n = 41), or both (n = 36). Household spread was noted in 5 cases (Appendix Table).

**Table:** Characteristics of the 157 proven cases of in an investigation of spread of antifungal-resistant *T. indotineae* infection, United Kingdom, 2017–2024*

Characteristics	No. (%), n = 157
Patient age range, y	
1–10	4 (2.5)
11–20	13 (8.3)
21–30	37 (23.6)
31–40	42 (26.8)
41–50	26 (16.6)
51–60	18 (11.5)
61–70	13 (8.3)
71–80	4 (2.5)
Anatomic site affected†	
Buttock, groin, gluteal fold, perineum, thigh	67 (42.7)
Back, abdomen, torso, trunk, breast, chest	18 (11.5)
Legs, feet, knee, toenail	14 (8.9)
Arms, hands, axilla	6 (3.8)
Face, neck, head	6 (3.8)
Unknown	53 (33.8)
Geographic location	
London	73 (46.5)
England outside London	54 (34.4)
Wales	8 (5.1)
Scotland	19 (12.1)
Republic of Ireland	3 (1.9)
Travel history‡	
Yes	41 (26.1)
No or unknown	116 (73.9)
Patient links to endemic area	
Yes	133 (84.7)
No	12 (7.6)
Unknown	12 (7.6)
Identification method	
Phenotypic only	114 (72.6)
Molecular ITS or WGS	43 (27.4)
Antifungal susceptibility testing	
Terbinafine, >0.5 mg/L	92 (58.6)
Terbinafine, <0.5mg/L	32 (20.4)
Terbinafine, not tested	33 (21.0)
Itraconazole, >05 mg/L	16 (10.2)
Itraconazole, <0.5 mg/L	92 (58.6)
Itraconazole, not tested	49 (31.2)

*Detailed case listings and definitions are provided (Appendix Table, https://wwwnc.cdc.gov/EID/article/31/1/24-0923-App1.pdf). †Multiple sites reported in some cases; therefore, total >157 cases. ‡Travel to India, Bangladesh, Pakistan, Sri Lanka, UAE, Nepal.

Before 2023, most (27/36) cases were identified in London, which was the most affected city according to total case numbers. Since 2023, increasing numbers of cases were found in an additional 27 cities in the United Kingdom and Ireland, and isolate numbers outside London exceed those in London (Appendix Figure 3). From 2018 to 2019, the prevalence of *T. indotineae* in the United Kingdom increased from 2% to 7% of all dermatophyte isolates referred to the MRL. This prevalence remained largely stable during 2019–2023 (range 5%–12%). Of note, *T. indotineae* comprised 38% of all dermatophyte isolates received by the MRL in 2024 up to July (Figure). 

**Figure:**
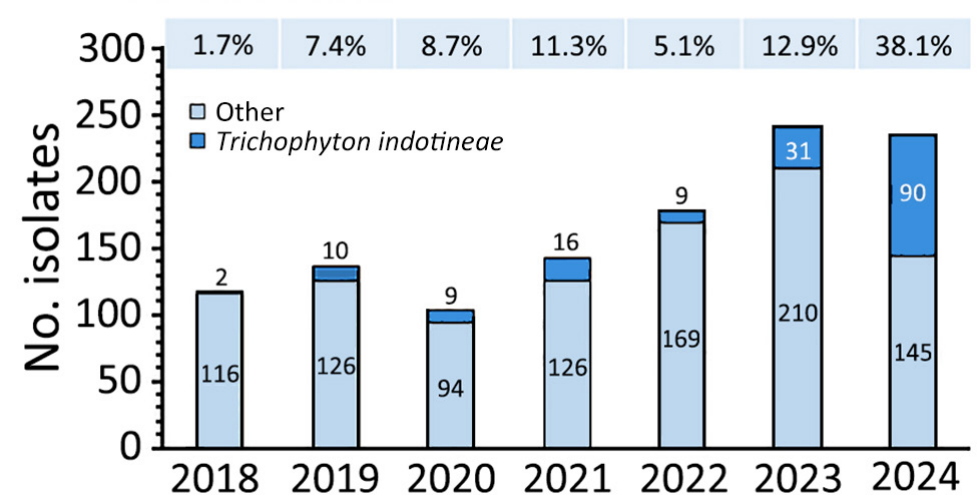
Numbers and percentages of isolates per year in study of spread of antifungal-resistant *Trichophyton indotineae*, United Kingdom, 2017–2024. Numbers of isolates of *T. indotineae* and all other dermatophyte species annually are referred to the UK National Mycology Reference Laboratory. Numbers above bars indicate percentages of all referrals that were *T. indotineae*.

Antifungal susceptibility data for terbinafine were available for 124/157 isolates, and in vitro resistance (MIC >0.5 mg/L) was documented in 92/124 (74.2%) cases, in keeping with previous reports (*1*,*2*,*4*,*5*). Of the 108 isolates in our study, 14% displayed MICs >0.5 mg/L to itraconazole; however, a breakpoint for itraconazole with *T. indotineae* is lacking. Fifty (31.8%) of 157 cases had documented treatment failure, 34 (21.7%) cases had terbinafine failure, and 7 (4.5%) cases had poor response to itraconazole.

In this study, London had the highest caseloads before 2023, likely because of absolute population numbers, comprehensive travel links to the Asian subcontinent through major London airports, and enhanced access to private dermatology clinics. The largely stable prevalence from 2019 through 2023 is probably because of COVID-19 prevention measures, which reduced population mixing and subsequent spread of *T. indotineae*. Our findings suggest that infections were acquired either directly in southern Asia and imported into the United Kingdom or from contacts with recent travel to such areas.

The first limitation of this study is underestimation of *T. indotineae* prevalence because of limited awareness among medical practitioners and microbiology laboratorians, likely misidentifications in routine laboratories, lack of commercial methods for rapid and accurate identification, and difficulties in obtaining skin scrapings from patients impeding laboratory identification of causative agent. Second, probable regional differences exist in awareness and identification capacity driven by regional prevalence and likelihood of prior encounter. Third, we do not have clinical information on dose or duration of terbinafine therapy for most patients with reported treatment failures; thus, we are unable to link treatment failure to elevated MIC values. Finally, only a proportion of *T. indotineae* isolates had genetic confirmation of identity. Despite our confidence in our methods, the identification of some cases by phenotypic methods alone could lead to some misidentification of species within the *T. mentagrophytes* species complex.

In conclusion, we show that *T. indotineae* was introduced into the United Kingdom from endemic areas and is spreading substantially. On the basis of current trends, we predict *T. indotineae* will rapidly become the predominant cause of tinea corporis in the United Kingdom. Clinicians and microbiology laboratorians should recognize this fungus as a predominant cause of tinea corporis.

Appendix Additional information on spread of antifungal-resistant *Trichophyton indotineae*, United Kingdom, 2017–2024.
