# Supplementary material for: Spread of Antifungal-Resistant Trichophyton indotineae, United Kingdom, 2017–2024
Source: Emerg Infect Dis. 2025 Jan;31(1):192–4. doi: 10.3201/eid3101.240923 (PMC11682803; doi:10.3201/eid3101.240923)
Supplement: Appendix — Additional information on spread of antifungal-resistant Trichophyton indotineae, United Kingdom, 2017–2024. [file 24-0923-Techapp-s1.pdf]

Article DOI: https://doi.org/10.3201/eid3101.240923

EID cannot ensure accessibility for supplementary materials supplied by authors. Readers who have difficulty accessing supplementary content should contact the authors for assistance.

Spread of Antifungal-Resistant *Trichophyton indotineae*, United Kingdom, 2017–2024

Appendix

Methods

Clinical Dermatophyte Isolates and Case Definitions

For isolate selection, we reviewed our laboratory electronic records. All dermatophyte isolates submitted to the UK Health Security Agency National Mycology Reference Laboratory between August 2017 and May 2024 for identification and/or antifungal susceptibility testing were included in this study. The majority of isolates originated from 3 centers in the United Kingdom: the National Mycology Reference Laboratory (MRL) in Bristol, southwest England; the Regional Mycology Reference Centre at Leeds Teaching Hospitals, northern England; and the Medical Microbiology Department at King's College Hospital, London, serving an ethnically diverse population in south and southeast London.

In "confirmed" cases (Appendix Table), dermatophytes were identified as *Trichophyton indotineae* by using a combination of molecular and/or phenotypic characteristics. For the additional 10 "likely" cases, we included dermatophyte isolates that were phenotypically identified as *Trichophyton mentagrophytes* complex but had increased terbinafine MIC causing tinea cruris/corporis and were isolated from chronic/recurrent infections. These isolates were not available for species-level identification, precluding formal confirmation that they were *T. indotineae*.

Data Collection

We collected patient demographic data (age range, ethnic background) from laboratory requisition forms submitted with clinical isolates. When available, we retrieved clinical and epidemiologic data including affected body site(s), disease duration, previous antifungal

treatment(s), ethnicity, and recent travel history from laboratory request forms or from conversations with referring physicians. All information on requisition forms was provided by requesting clinicians as part of the routine standard of care for their patients. In this study, we considered *T. indotineae* infection endemic to the Indian subcontinent (*1*). A link to the endemic area was defined as South-Asian ethnicity.

Phenotypic Identification

All dermatophyte isolates received at MRL were initially subcultured onto Sabouraud glucose peptone agar supplemented with chloramphenicol (Oxoid) and incubated at 28°C–30°C for 7–14 days before identification. Cultures were examined for macroscopic features and microscopic characteristics. For identification of *T. indotineae*, the presence of abundant fusiform to clavate, thin and smooth-walled macroconidia measuring 6–8 × 20–50 µm with 3–5 septa and an acute apical tip was used as a key defining feature (Appendix Figure 1). Some macroconidia showed narrow attachment bases. Occasionally, shorter club-shaped macroconidia were present. In addition, isolates identified as *T. indotineae* displayed clusters of spherical microconidia arranged around differentiated hyphae. Numerous subspherical and pyriform microconidia were along undifferentiated hyphae. Spiral hyphae and chlamydoconidia (single or in chains) were present in some cultures.

Colonies of *T. indotineae* were flat with a granular, powdery to floccose texture. Most isolates showed a fast to moderate growth rate. Surface of colonies remained white, beige, or suede-like in color. Reverse pigmentation was variable, and most isolates displayed light brown, cream, or yellow colors (Appendix Figure 2).

Internal Transcribed Spacer (ITS) Sequencing

Fungal DNA extraction, PCR amplification and sequencing of the ITS1 region and BLASTN alignments against sequences in public reference databases were performed exactly as previously described (*2*). All ITS1 sequences generated in this study were identical to each other and shared 100% homology with reference *Trichophyton indotineae* sequences in the public databases including the sequence for the type strain LC508024. A representative ITS1 sequence from the current study was deposited in GenBank under accession no. PQ279401.

Antifungal Susceptibility Testing

Terbinafine and itraconazole antifungal susceptibility testing was determined according to the CLSI M38-A2 broth microdilution method (*3*). All isolates were initially subcultured onto Sabouraud glucose peptone agar supplemented with chloramphenicol (Oxoid) and incubated at 28°C–30°C for 7–14 days before antifungal susceptibility testing. Antifungal drugs were obtained from their respective manufacturers as standard powders. To prepare stock solutions, terbinafine (Sigma Chemical Co.) was dissolved in dimethyl sulfoxide (DMSO). Itraconazole powder (Janssen Research Foundation) was dissolved in PEG400 by heating at 70°C. Serial 2-fold dilutions of both drugs were prepared in RPMI 1640 (Sigma Chemical Co.) buffered with 0.165 M MOPS with 0.2% glucose and phenol red, without bicarbonate. Final testing concentrations were 0.03 to 16 mg/L for both terbinafine and itraconazole. MICs were read at 80% inhibition of growth compared with the drug-free growth control after ≥96 hours of incubation. All assays included the control *Aspergillus fumigatus* strains NCPF 7097 and NCPF7100. In the absence of CLSI-established clinical breakpoint for terbinafine, we adapted tentative MIC value of ≥0.5 mg/L to identify non–wild-type (WT) isolates.

Whole-Genome Sequencing and Analysis

Genomic DNA (gDNA) was extracted as previously described (*4*). Briefly, fungal isolates were subcultured on Sabauroud glucose agar (SGA) plates supplemented with chloramphenicol and incubated at 28°C –30°C for 7–10 days. Stock conidial suspensions were prepared by washing the surface of the SGA plates with 10 mL of sterile water containing 0.05% Tween 20. The conidial suspensions were filtered by using Miracloth (EMD Chemicals) to remove fungal hyphae, transferred to 50-mL sterile conical tubes, and centrifuged at maximum speed (10,000 × *g*) for 10 minutes. The supernatants were discarded, and the pellets were resuspended in 5 mL of sterile distilled water. The concentrations of the suspended conidial stocks were determined by counting the conidia by using a hemocytometer chamber at ×400 magnification. Harvested conidia at concentrations of 2×10^8/mL were subjected to DNA extraction. High-molecular-weight DNA was extracted with an optimized MasterPure Complete DNA and RNA purification kit (Lucigen) with an additional bead-beating step included. Harvested conidia were homogenized by using 1.0-mm-diameter zirconia/silica beads (BioSpec Products) in a FastPrep-24 system (MP Biomedicals) at 4.5 m/s for 45 seconds. After a purification and concentration step using a DNeasy Blood and Tissue kit (Qiagen), gDNA was

quantified by using a Qubit 2.0 fluorometer and dsDNA BR (double-stranded DNA, broad-range) assay kit (Life Technologies). Quality control of extracted gDNA samples before library preparation was performed by using the TapeStation 2200 system (Agilent) and gDNA ScreenTape assays. gDNA libraries were constructed, normalized, and indexed at Earlham Institute and run on a NovaSeq 6000 SP v1.5 flow cell to generate 150-bp paired-end reads.

Whole-genome data were analyzed at Imperial College London, United Kingdom, as part of a multicenter international study. In brief, a custom bioinformatics pipeline was used to analyze the sequencing data. The bioinformatics pipeline included first mapping the raw reads to the *T. indotineae* reference genome (GenBank GCA_023065905.1; strain TIMM20114) by using the Burrows Wheeler Aligner (BWA) MEM algorithm v0.7.17 (H. Li, unpub. data). All raw genomic data are available under the Project Accession no. PRJEB75499.

References

1. Ebert A, Monod M, Salamin K, Burmester A, Uhrlaß S, Wiegand C, et al. Alarming India-wide phenomenon of antifungal resistance in dermatophytes: a multicentre study. Mycoses. 2020;63:717–28. PubMed https://doi.org/10.1111/myc.13091

2. Borman AM, Desnos-Ollivier M, Campbell CK, Bridge PD, Dannaoui E, Johnson EM. Novel taxa associated with human fungal black-grain Mycetomas: *Emarellia grisea* gen. nov., sp. nov., and *Emarellia paragrisea* sp. nov. J Clin Microbiol. 2016;54:1738–45. PubMed https://doi.org/10.1128/JCM.00477-16

3. Clinical and Laboratory Standards Institute. Reference method for broth dilution antifungal susceptibility testing of filamentous fungi (C38), 3rd edition. Wayne (PA): The Institute; 2017.

4. Shelton JMG, Rhodes J, Uzzell CB, Hemmings S, Brackin AP, Sewell TR, et al. Citizen science reveals landscape-scale exposures to multiazole-resistant *Aspergillus fumigatus* bioaerosols. Sci Adv. 2023;9:eadh8839. PubMed https://doi.org/10.1126/sciadv.adh8839

Appendix Table. Clinical details for isolates of *Trichophyton indotineae**

Isolate no.	Sample date	Age, y	Sample site	Location	Clinical history	Link to endemic area†	Recent travel	TERB MIC, mg/L	ITR MIC, mg/L	Identification method
Confirmed isolates										
1	10.09.2018	31–40	Buttock	London	NA	Yes	India	**1.0**	—	Phenotypic/WGS
2	14.01.2019	41–50	Groin	London	NA	Yes	India	**4.0**	—	Phenotypic/WGS
3	17.01.2019	51–60	Back	London	10-mo intractable tinea corporis, no response to itraconazole, itraconazole and terbinafine combination	Yes	India	**>16.0**	—	Phenotypic/WGS
4	04.02.2019	51–60	Torso	London	NA	Yes	India	**4.0**	—	Phenotypic/WGS
5	16.05.2019	31–40	Groin	London	NA	No		<0.03	—	Phenotypic/WGS
6	03.10.2019	51–60	Buttock	London	6-mo rash, high dose prednisolone	Yes		0.125	—	Phenotypic/WGS
7	20.01.2020	51–60	Groin	London	3mo rash	Yes		0.06	—	Phenotypic/WGS
8	21.02.2020	61–70	Groin	London	Rash	Yes		**2.0**	—	Phenotypic/WGS
9	11.11.2020	1–10	Left arm	London	8-mo tinea corporis, no improvement with daktacort, elocon, terbinafine, canesten, locoid	Yes	Bangladesh	**8.0**	—	Phenotypic/WGS
10	09.12.2020	1–10	Right leg, foot	London	NA	Yes	UAE	**2.0**	—	Phenotypic/WGS
11	23.12.2020	31–40	Buttock, groin	London	NA	Yes	Sri Lanka	**2.0**	—	Phenotypic/WGS
12	08.01.2021	41–50	Unknown skin	London	NA	Yes		0.125	0.125	Phenotypic/WGS/ITS
13	12.02.2021	41–50	Groin	London	6-mo extending scaly rash, well demarcated	Yes	Bangladesh	**2.0**	—	Phenotypic/WGS
14	26.02.2021	41–50	Buttock	London	Tinea incognito, widespread confluent annular lesions	Yes	India	**2.0**	—	Phenotypic/WGS
15	08.03.2021	41–50	Axilla	London	Persistent axillar rash	Yes	India	**4.0**	—	Phenotypic/WGS
16	01.04.2021	21–30	Leg	Dublin	NA	Yes		**4.0**	0.06	Phenotypic/WGS
17	03.04.2021	41–50	Thigh	London	>1-y scaly erythematous lesions, response to itraconazole but recurred	Yes	India	**8.0**	—	Phenotypic/WGS
18	21.05.2021	1–10	Left arm	London	Annular rash	Yes		**8.0**	0.125	Phenotypic/WGS
19	26.05.2021	61–70	Unknown skin	London	NA	Unknown		0.03	0.125	Phenotypic/WGS
20	03.06.2021	21–30	Groin	Oxford	3-mo rash, no response topical and oral terbinafine	Yes		**4.0**	—	Phenotypic/WGS
21	07.07.2021	31–40	Unknown skin	London	Failed 2 courses oral terbinafine over 3 mo	Yes		**8.0**	0.25	Phenotypic/WGS
22‡	26.07.2021	11–20	Unknown skin	Leeds	Terbinafine-resistant tinea corporis	Yes	India	**2.0**	0.125	Phenotypic/WGS/ITS
23	20.08.2021	71–80	Unknown nail	Leeds	Terbinafine failure	Unknown		**1.0**	<0.03	Phenotypic/WGS
24	17.09.2021	71–80	Unknown nail	Edinburgh	NA	Unknown		**2.0**	0.25	Phenotypic/WGS
25	07.10.2021	31–40	Unknown skin	London	Recalcitrant tinea	Yes		**4.0**	0.06	Phenotypic/WGS

Isolate no.	Sample date	Age, y	Sample site	Location	Clinical history	Link to endemic area†	Recent travel	TERB MIC, mg/L	ITR MIC, mg/L	Identification method
26	25.10.2021	21–30	Unknown skin	London	NA	Yes	India	**2.0**	—	Phenotypic/WGS
27	24.11.2021	61–70	Torso	London	3-y history of widespread rash	Yes		**1.0**	0.5	Phenotypic/WGS
28	01.02.2022	41–50	Unknown nail	Liverpool	NA	Unknown		**2.0**	0.5	Phenotypic
29	18.02.2022	21–30	Unknown skin	London	Tinea corporis	Yes		**2.0**	0.25	Phenotypic
30	17.03.2022	21–30	Groin, legs	Leeds	Annular rash, partner traveled to India	Yes	India	**2.0**	0.06	Phenotypic/ITS
31	29.03.2022	21–30	Abdomen	Leeds	Extensive hyperpigmented rash on abdomen	Yes		<0.03	0.25	Phenotypic/ITS
32	31.05.2022	31–40	Buttock	London	NA	Yes	India	<0.03	0.125	Phenotypic/WGS
33	06.09.2022	31–40	Abdomen	Edinburgh	NA	Yes		**4.0**	0.06	Phenotypic
34	03.10.2022	11–20	Genitals, face	Leeds	Failed terbinafine and fluconazole	Yes		**4.0**	0.06	Phenotypic/ITS
35	22.11.2022	31–40	Groin	Leeds	Tinea cruris, pregnant on topical treatment	Yes		<0.03	<0.03	Phenotypic/ITS
36	25.11.2022	21–30	Gluteal fold	London	Tinea cruris now extensive tinea corporis, failed terbinafine, partial response to itraconazole	Yes	India	0.06	<0.03	Phenotypic
37	25.01.2023	31–40	Back	Leeds	NA	Yes		<0.03	0.25	Phenotypic/ITS
38	03.04.2023	21–30	Groin	Leeds	Tinea cruris	Yes		**1.0**	0.125	Phenotypic/ITS
39	13.06.2023	11–20	Unknown skin	London	NA	Yes		**0.5**	<0.03	Phenotypic
40	29.06.2023	51–60	Groin	Leeds	Annular eruption groin, umbilicus, sub-mammary, abdomen	Yes		—	0.25	Phenotypic/ITS
41	17.07.2023	21–30	Buttock	London	3-y history of rash, no response to antifungals	Yes	Nepal	**2.0**	0.06	Phenotypic/ITS
42	21.07.2023	61–70	Finger	London	NA	Yes		—	—	Phenotypic
43	01.08.2023	21–30	Thighs	Glasgow	Fungal infection both inner thighs, not responding	Yes	India	**2.0**	0.06	Phenotypic
44	02.08.2023	31–40	Groin	Leeds	Tinea cruris, recent travel	Unknown	Bangladesh	**0.5**	<0.03	Phenotypic
45	14.08.2023	21–30	Unknown tissue	London	NA	Yes		—	—	Phenotypic
46	06.09.2023	61–70	Groin	London	Rash in groin	Yes		<0.03	0.06	Phenotypic
47	07.09.2023	21–30	Thigh	Glasgow	Tinea corporis involving thighs	Yes		**2.0**	0.06	Phenotypic
48	10.09.2023	21–30	Groin	Durham	NA	Yes		—	—	Phenotypic
49	10.10.2023	71–80	Unknown skin	Coventry	Itchy rash, no response to 2.5 mo of terbinafine	Unknown	India	**0.5**	0.125	Phenotypic
50	17.10.2023	11–20	Back	Leeds	Scaly rash upper back for 10 mo, parents have similar	Yes		**0.5**	<0.03	Phenotypic
51	20.10.2023	21–30	Right Leg	London	Progressive extensive tinea for >6 mo, minimal response to terbinafine	No	South America	**2.0**	0.25	Phenotypic

Isolate no.	Sample date	Age, y	Sample site	Location	Clinical history	Link to endemic area†	Recent travel	TERB MIC, mg/L	ITR MIC, mg/L	Identification method
52	25.10.2023	21–30	Unknown skin	Leeds	Tinea cruris not responding to terbinafine	Yes		**2.0**	0.25	Phenotypic/ ITS
53	26.10.2023	51–60	Unknown skin	Blackpool	Rash	Yes	Bangladesh	—	—	Phenotypic/ ITS
54	30.10.2023	21–30	Unknown	Coventry	Antifungal resistant tinea	Yes		**0.5**	0.25	Phenotypic
55	30.10.2023	31–40	Leg	Leeds	Tinea	Yes		**1.0**	0.25	Phenotypic
56	07.11.2023	41–50	Perineum	Cardiff	Itchy rash, no response to terbinafine	Yes	India	0.25	<0.03	Phenotypic
57	14.11.2023	31–40	Groin	Edinburgh	Recurrent tinea	Unknown		**4.0**	0.125	Phenotypic
58	14.11.2023	31–40	Groin	Edinburgh	Recurrent thrush	Unknown		**4.0**	0.125	Phenotypic
59	16.11.2023	31–40	Groin	Edinburgh	NA	Unknown		**2.0**	0.25	Phenotypic/ ITS
60	18.11.2023	51–60	Unknown skin	London	Fungal skin infection	Yes		—	—	Phenotypic
61‡	21.11.2023	11–20	Groin	Leeds	Recurrent tinea cruris	Yes	India	**1.0**	0.06	Phenotypic
62	06.12.2023	51–60	Groin	Blackpool	Tinea cruris	Yes	Bangladesh	—	—	Phenotypic
63	06.12.2023	41–50	Buttock	Cardiff	Rash, no response to topical terbinafine	Yes		0.25	<0.03	Phenotypic
64	13.12.2023	41–50	Thigh	Glasgow	Tinea cruris, children same, failed 2 courses of terbinafine	Yes		**2.0**	0.25	Phenotypic
65	18.12.2023	31–40	Abdomen	Southampton	Tinea of abdomen and arm	Yes		**0.5**	0.125	Phenotypic
66	19.12.2023	21–30	Knee	Leeds	Extensive tinea cruris and corporis	Yes		**2.0**	0.06	Phenotypic
67	28.12.2023	41–50	Chest	Leeds	Rash on forearm and chest	Unknown	Bangladesh	**0.5**	0.06	Phenotypic/ ITS
68	02.01.2024	11–20	Abdomen	Bristol	Ongoing skin rash	Yes		—	—	Phenotypic/ ITS
69	09.01.2024	61–70	Groin	Newcastle	1-y history tinea cruris, no response to 3 mo of terbinafine, partial response to itraconazole	Yes	Pakistan	—	0.125	Phenotypic
70	17.01.2024	41–50	Thigh	London	Recurrent tinea corporis, no response to antifungals	Yes		**0.5**	0.25	Phenotypic
71	19.01.2024	31–40	Unknown skin	Glasgow	Fungal skin infection, not resolved with oral terbinafine	Yes		<0.03	0.125	Phenotypic
72	31.01.2024	51–60	Groin	Cambridge	NA	No		—	—	Phenotypic
73	01.02.2024	11–20	Thigh skin biopsy	Poole	Fungal rash	No		—	—	Phenotypic
74	05.02.2024	21–30	Leg	London	Widespread scaly lesions on legs	Yes		—	—	Phenotypic
75	07.02.202 2	31–40	Unknown skin	London	Skin infection not responding to antifungals	Yes		<0.03	0.125	Phenotypic
76	16.02.2024	41–50	Buttock	Glasgow	Tinea cruris, multi-drug resistant	Yes		**1.0**	0.5	Phenotypic
77	20.02.2024	51–60	Unknown skin	London	Fungal rash on body	Yes		—	—	Phenotypic
78	22.02.2024	51–60	Unknown	Glasgow	Severe/widespread dermatophyte	Yes		**1.0**	0.06	Phenotypic/ ITS

Isolate no.	Sample date	Age, y	Sample site	Location	Clinical history	Link to endemic area†	Recent travel	TERB MIC, mg/L	ITR MIC, mg/L	Identification method
					infection, terbinafine failure					
79	22.02.2024	41–50	Unknown skin	London	Extensive tinea corporis	Yes		**1.0**	0.125	Phenotypic
80	23.02.2024	21–30	Unknown skin	London	Resistant tinea corporis, no response to 6w oral terbinafine	Yes		**1.0**	0.06	Phenotypic
81	26.02.2024	51–60	Groin	Glasgow	Tinea cruris	Yes		<0.03	0.06	Phenotypic
82	26.02.2024	41–50	Thigh	London	Recurrent tinea corporis, not responding to antifungals	Yes		**0.5**	0.25	Phenotypic
83	27.02.2024	31–40	Buttock	London	Persistent tinea of buttocks despite 6w oral terbinafine	Yes		**1.0**	0.06	Phenotypic
84	27.02.2024	21–30	Thigh	Ireland	Extensive tinea corporis involving groin and thighs now spread to hands and face. No response to 6 wk of antifungals	Yes	Bangladesh	**0.5**	0.25	Phenotypic
85	01.03.2024	31–40	Abdomen	Bristol	Large annular patches groin and abdomen	Unknown		—	—	Phenotypic/ ITS
86	04.03.2024	61–70	Groin	Glasgow	5-y history of treatment-resistant pruritic rash to the groin	Yes		<0.03	<0.03	Phenotypic
87	05.03.2024	41–50	Unknown skin	Glasgow	Widespread tinea corporis	Yes		0.125	<0.03	Phenotypic
88	07.03.2024	41–50	Groin	London	Recalcitrant tinea corporis	Yes		**1.0**	0.5	Phenotypic
89	11.03.2024	21–30	Buttocks	Cardiff	Persistent tinea of buttocks for 2 y, incomplete response to fluconazole and miconazole	Yes		**1.0**	0.125	Phenotypic
90	12.03.2024	61–70	Groin/Thigh skin	Warwick	Dermatitis affecting groin and upper thigh not responding to treatment	Yes		**1.0**	0.25	Phenotypic
91	13.03.2024	21–30	Unknown skin	London	Widespread tinea corporis	Yes	Bangladesh	0.03	0.06	Phenotypic
92	14.03.2024	31–40	Unknown skin	London	Ringworm, no response to terbinafine and itraconazole; partner also has lesions	Yes	Bangladesh	—	—	Phenotypic
93	18.03.2024	31–40	Buttock	Glasgow	Large patch of ringworm on buttock despite canesten treatment	Yes		<0.03	0.06	Phenotypic
94	18.3.2024	11–20	Legs	London	Tinea incognito	Yes		—	—	Phenotypic
95	19.03.2024	31–40	Thigh	London	Tinea corporis affecting thighs	Yes		—	—	Phenotypic
96	22.03.2024	11–20	Unknown skin	Durham	Large eruption on lower abdomen for 1 y, not responding to antifungal treatment	Yes		<0.03	0.06	Phenotypic
97	26.03.2024	01–10	Head	London	Persistent scaling on head, tinea	Yes		—	—	Phenotypic
98	04.04.2024	31–40	Groin	London	Tinea cruris	Yes		**2**	0.5	Phenotypic

Isolate no.	Sample date	Age, y	Sample site	Location	Clinical history	Link to endemic area†	Recent travel	TERB MIC, mg/L	ITR MIC, mg/L	Identification method
99	04.04.2024	61–70	Back	Bristol	Fungal rash since travel to India, not responding to clotrimazole, terbinafine or itraconazole	No	India	**2**	1.0	Phenotypic
100	05.04.2024	41–50	Abdomen	London	4.5-y recalcitrant tinea corporis/cruris affecting abdomen, legs, buttocks. Repeated oral and topical treatment (incl. terbinafine) failures	Unknown		**1.0**	<0.03	Phenotypic
101	05.04.2024	41–50	Groin	Bristol	1-y history of tinea cruris now involving axilla, no response to topical terbinafine, partial response to itraconazole	Yes	India	—	—	Phenotypic
102	05.04.2024	11–20	Foot	London	NA	Yes		—	—	Phenotypic
103	09.04.2024	31–40	Wrist	London	Scaly patch on wrist	No		0.125	0.06	Phenotypic
104	11.04.2024	21–30	Unknown skin	Newcastle	Widespread rash for 2.5 y, not responding to multiple topical treatments including terbinafine	Yes	India	**1.0**	<0.03	Phenotypic
105	12.04.2024	11–20	Trunk	Bristol	Spreading rash for 5 mo, no response to 14 d of terbinafine	Yes		—	—	Phenotypic
106	12.04.2024	61–70	Unknown skin	Southampton	Rash, all family members affected	Yes		—	—	Phenotypic
107	13.04.2024	31–40	Thigh	London	Tinea incognito involving gluteus, thighs, and upper arm	Yes		**0.5**	0.06	Phenotypic
108	16.04.2024	21–30	Thigh	London	NA	Yes		**2.0**	<0.03	Phenotypic
109	17.04.2024	31–40	Unknown skin	London	NA	Yes		—	—	Phenotypic
110	19.04.2024	31–40	Toenail	London	NA	Yes		—	—	Phenotypic
111	19.04.2024	21–30	Groin	London	Tinea cruris	Yes		—	—	Phenotypic
112	23.04.2024	31–40	Thigh skin	Birmingham	Thigh lesions, terbinafine-resistant treatment failure	Yes		**0.5**	0.125	Phenotypic
113	24.04.2024	41–50	Thigh	Glasgow	NA	Yes		0.25	0.25	Phenotypic
114	25.04.2024	41–50	Unknown skin	Newcastle	Multiple annular rashes	Yes	Bangladesh	**0.5**	0.125	Phenotypic
115	27.04.2024	21–30	Thigh	London	Recurrent inner thigh infection	Yes		—	—	Phenotypic
116	02.05.2024	21–30	Skin back	London	Fungal infection involving buttocks and back, resistant to terbinafine	Yes		**1.0**	1.0	Phenotypic
117	14.05.2024	21–30	Unknown skin	Edinburgh	Resistant fungal infection	Yes		**1.0**	0.25	Phenotypic
118	15.05.2024	51–60	Unknown skin	London	Rash	Yes		**1.0**	0.25	Phenotypic
119	17.05.2024	41–50	Unknown skin	London	Rash	Yes		**4.0**	1.0	Phenotypic
120	20.05.2024	21–30	Forehead	Cornwall	Itchy rash, ringworm/kerion	No		—	—	Phenotypic

Isolate no.	Sample date	Age, y	Sample site	Location	Clinical history	Link to endemic area†	Recent travel	TERB MIC, mg/L	ITR MIC, mg/L	Identification method
121	20.05.2024	21–30	Unknown skin	London	Extensive tinea, now on fluconazole as resistance concerns	Yes		**2**	0.125	Phenotypic
122	20.05.2024	21–30	Unknown skin	London	Fungal rash on body	Yes		**4.0**	0.25	Phenotypic
123	22.05.2024	51–60	Groin, wrist	Somerset	Skin rash	Yes	India	—	—	Phenotypic
124	23.05.2024	31–40	Unknown	Cardiff	3-y tinea corporis	Yes		**2.0**	0.5	Phenotypic
125	24.05.2024	41–50	Groin	London	NA	Yes		**4.0**	0.125	Phenotypic
126	28.05.2024	11–20	Buttock	London	Tinea corporis affecting buttocks	Yes	Bangladesh	**4.0**	0.25	Phenotypic
127	08.06.2024	61–70	Unknown skin	London	Tinea corporis	Yes		**2.0**	0.5	Phenotypic
128	10.06.2024	71–80	Unknown tissue	Ireland	None given	No		—	—	Phenotypic
129	13.06.2024	31–40	Unknown skin	Cardiff	Tinea corporis lower legs buttocks, no response to 4 wk of oral and topical terbinafine	Yes		**2.0**	<0.03	Phenotypic
130	20.06.2024	21–30	Leg/neck	Middlesborough	Skin infection, treatment failure	Yes		**2.0**	0.25	Phenotypic
131	24.06.2024	31–40	Legs, buttocks	Leeds	Tinea lesions	Yes		<0.03	<0.03	Phenotypic
132	26.06.2024	21–30	Unknown skin	London	Scaly lesions, not responding to topical treatments	Yes		<0.03	0.25	Phenotypic
133	27.06.2024	31–40	Unknown skin	London	NA	Yes		0.06	0.25	Phenotypic
134	27.06.2024	41–50	Skin	Leeds	Annular scaly rash buttocks, back groin and abdomen	Yes		**1.0**	0.06	Phenotypic
135	28.06.2024	51–60	Unknown	Coventry	NA	Yes		**1.0**	0.125	Phenotypic
136	01.07.2024	31–40	Unknown	Coventry	NA	Yes		—	—	Phenotypic
137	01.07.2024	41–50	Unknown	London	9-mo history of dermatophyte infection	Yes		**0.5**	1.0	Phenotypic
138	08.07.2024	51–60	Foot	Milton Keynes	Diabetic surgical wound	Yes		—	—	Phenotypic
139	08.07.2024	11–20	Breast	Leeds	8-mo intermittent scaly rash left breast, had used steroid antifungal cream	Yes	India	0.125	0.25	Phenotypic
140	10.07.2024	21–30	Unknown skin	London	5-mo history of rash post travel	Yes	Bangladesh	**4.0**	0.5	Phenotypic
141	11.07.2024	31–40	Unknown	London	Persistent fungal rash	Yes		**2.0**	0.125	Phenotypic
142	15.07.2024	41–50	Nail	Bournemouth	Post chemotherapy	No		0.125	—	Phenotypic
143	16.07.2024	21–30	Leg	Leeds	NA	Yes		<0.03	<0.03	Phenotypic
144	16.07.2024	31–40	Unknown skin	Cardiff	NA	Yes		**1.0**	0.25	Phenotypic
145	17.07.2024	61–70	Buttock	Warwick	NA	Yes		**2.0**	0.125	Phenotypic
146	18.07.2024	31–40	Buttocks/face	Cardiff	Tinea corporis for 6 mo, not cleared after 2 × 1 mo oral terbinafine	Yes		**2.0**	0.25	Phenotypic
147	19.07.2024	31–40	Chin	Warwick	Fungal rash	No		—	—	Phenotypic

Isolate no.	Sample date	Age, y	Sample site	Location	Clinical history	Link to endemic area†	Recent travel	TERB MIC, mg/L	ITR MIC, mg/L	Identification method
148	25.07.2024	61–70	Groin/abdomen	Leeds	Tinea	Yes		<0.03	0.06	Phenotypic
149	25.07.2024	31–40	Groin swab	London	Tinea cruris with scaly rash	Yes		0.125	0.125	Phenotypic
150	27.07.2024	51–60	Groin	London	No improvement with fluconazole, terbinafine, miconazole	Yes	Pakistan	**1.0**	0.5	Phenotypic
151	30.07.2024	31–40	Unknown skin	London	Tinea corporis, not responding to terbinafine	No		**4.0**	2.0	Phenotypic
152	30.07.2024	21–30	Buttock	Leeds	9 mo of itraconazole and steroids	Yes	Pakistan	**1.0**	0.25	Phenotypic
153	31.07.2024	21–30	Unknown skin	London	Terbinafine unresponsive	Yes		**2.0**	0.25	Phenotypic
154	02.08.2024	31–40	Back	Glasgow	No improvement on oral terbinafine	Yes	Bangladesh	**2.0**	0.5	Phenotypic
155	14.08.2024	51–60	Thigh	Glasgow	5-mo rash not responding to topical antifungals or oral fluconazole	Yes		**2.0**	0.5	Phenotypic
156	16.08.2024	31–40	Unknown skin	Cardiff	Fungal rash	Yes		0.06	0.25	Phenotypic
157	04.09.2024	31–40	Groin	Bristol	Tinea cruris failed to respond to 2 mo of terbinafine, spreading to legs	No	Iran	—	—	Phenotypic
Additional likely isolates										
1	07.08.2017	31–40	Groin	London	Tinea cruris	Unknown		**16.0**	<0.03	Provisional identification
2	31.12.2018	51–60	Legs	London	Deep infiltrative nodules on legs	Yes		**4.0**	0.125	Provisional identification
3	27.02.2019	51–60	Back	London	Tinea corporis of back, no response to terbinafine	Yes		**>16.0**	1.0	Provisional identification
4	27.02.2019	61–70	Arm	London	Tinea corporis of arm	No		**8.0**	0.5	Provisional identification
5	27.03.2019	21–30	Thigh	London	Recurrent tinea cruris	Yes		**2.0**	0.5	Provisional identification
6	16.12.2019	11–20	Unknown skin	Oxford	18-mo history of treatment-resistant tinea corporis	Unknown		**1.0**	0.5	Provisional identification
7	15.01.2020	11–20	Abdomen	Norwich	Extensive tinea corporis	Yes		**2.0**	0.5	Provisional identification
8	17.01.2020	51–60	Unknown skin	Sheffield	Chronic tinea	No		**4.0**	0.5	Provisional identification
9	21.02.2020	61–70	Groin	London	Groin fungal infection	Yes		**2.0**	0.25	Provisional identification
10	12.08.2020	61–70	Unknown skin	London	Deep infiltrative nodules on legs	Yes		**2.0**	0.125	Provisional identification

*Bold MIC values for terbinafine are equal to or higher than the suggested clinical break point (0.5 mg/L). ITR, itraconazole; ITS, internal transcribed spacer; NA, not available; TERB, terbinafine; WGS, whole-genome sequencing; —, not tested.
†Link to endemic area was defined as South Asian ethnic background (Appendix).
‡Isolates 22 and 61 were collected from the same patient 2 years apart.

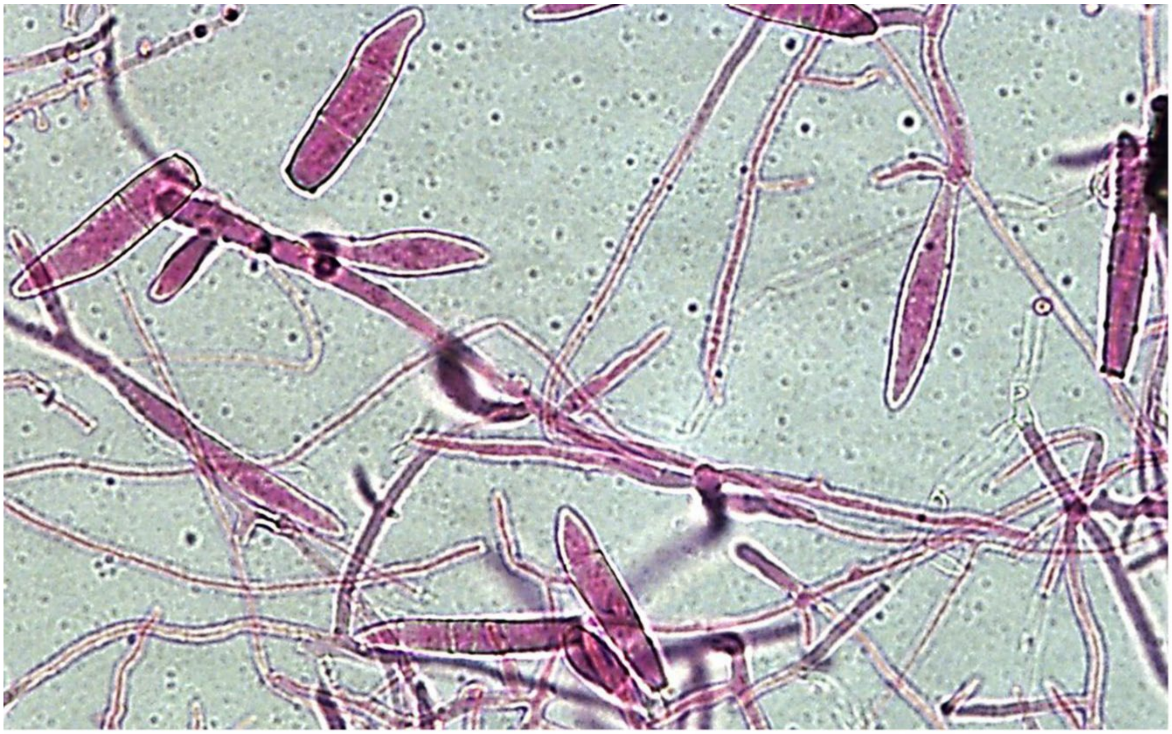

Appendix Figure 1. Microscopic feature of *Trichophyton indotineae* macroconidia, United Kingdom, 2017–2024. Sellotape preparation stained with lactofucshin (original magnification, ×400).

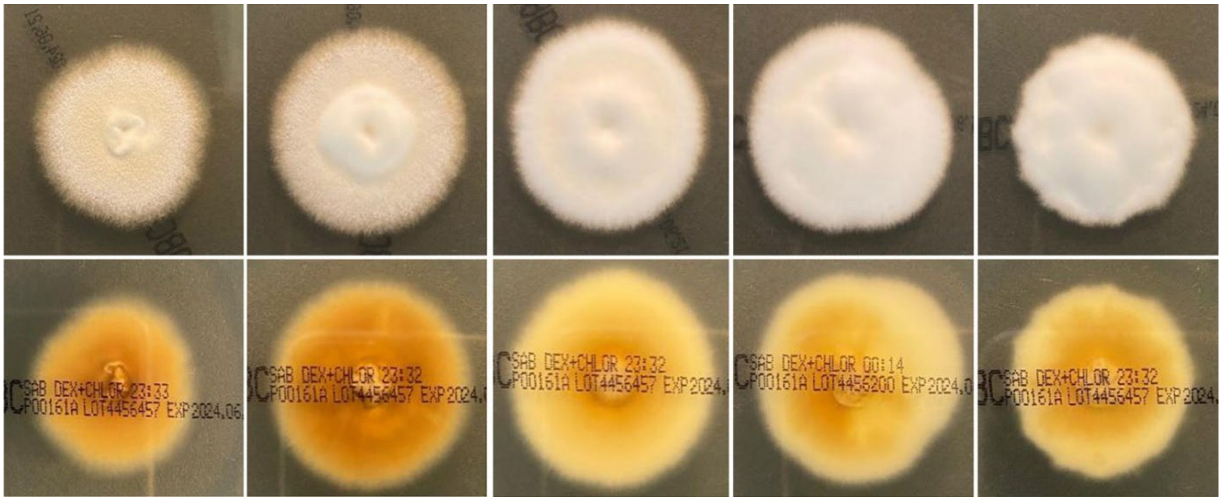

Appendix Figure 2. Macroscopic characteristics of 5 clinical isolates of *Trichophyton indotineae*, United Kingdom, 2017–2024. Top row, surface; bottom row, reverse of the same colony after a 14-day incubation at 28°C–30°C.

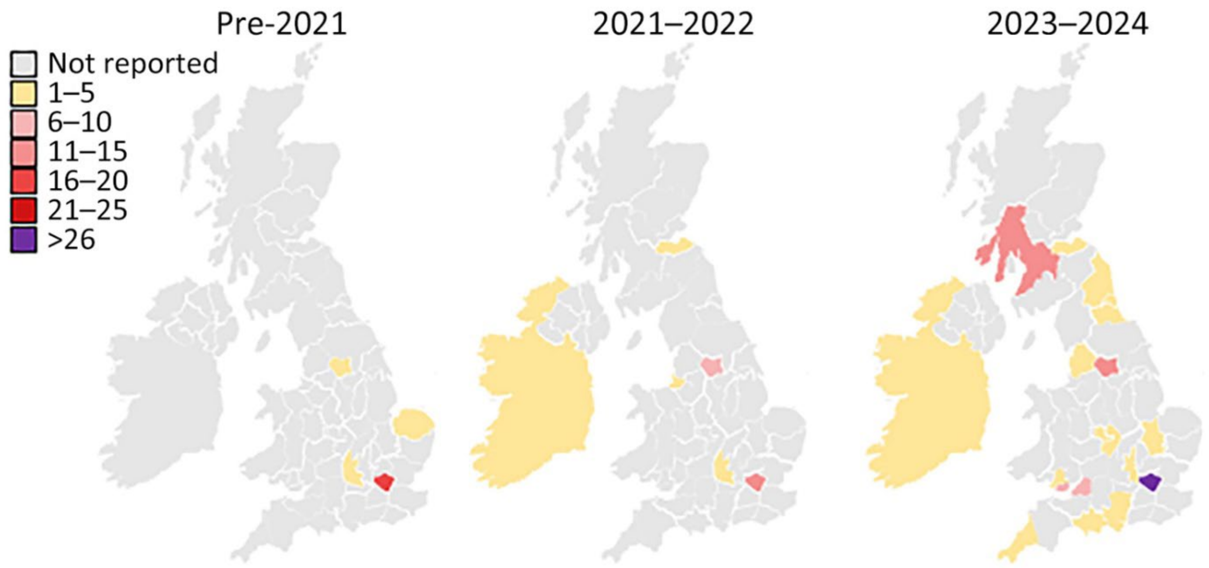

Appendix Figure 3. Geographic distribution and numbers of cases of *T. indotineae* across the United Kingdom at various time points between 2017 and mid-2024. Data for the 157 proven and 10 additional likely cases included here are provided in the Appendix Table.
